# Cytokine profiles in highly active antiretroviral treatment non-adherent, adherent and naive HIV-1 infected patients in Western Kenya

**DOI:** 10.4314/ahs.v21i4.12

**Published:** 2021-12

**Authors:** Fauzia Musa, Nathan Shaviya, Fidelis Mambo, Collins Abonyo, Erick Barasa, Philemon Wafula, George Sowayi, Mustafa Barasa, Tom Were

**Affiliations:** 1 Kakamega Provincial General Hospital; 2 Masinde Muliro University of Science and Technology, Medical Laboratory Sciences; 3 Masinde Muliro University of Science and Technology; 4 Kenya Ministry of Health; 5 Masinde Muliro University of Science and Technology, Biological Sciences

**Keywords:** Cytokines, non-adherence, HAART

## Abstract

**Background:**

Cytokines play an important role in signaling the immune system to build an adequate immune response against HIV. HIV distorts the balance between pro and anti-inflammatory cytokines causing viral replication. Highly active antiretroviral treatment (HAART) acts by trying to restore pro and anti-inflammatory cytokine balance. It is not clear how HAART non-adherence influences circulating cytokine levels. This study therefore determined cytokine levels in HAART non-adherent individuals.

**Methods:**

This cross-sectional study recruited 163 participants (51 controls, 23 HIV-1+ HAART naive, 28 HAART-adherent 6 months, 19 HAART-adherent 12 months and 42 HAART non-adherent). Cytokines were analyzed by ELISA while CD4 T cells determined in 3.0 µl of whole blood using BD FACSCaliburTM and viral load in 0.2ml plasma sample using Abbott Molecular m2000sp sample preparation and m2000rt real-time amplification and detection systems (Abbott Molecular Inc., Illinois, USA) according to the manufacturer's methods.

**Results:**

IL-4, IL-6, IL-10, TNF-α and TGF-β were significantly elevated in HIV-1 HAART non-adherent compared with HIV-1 HAART adherent and healthy controls P<0.01. IFN- γ was significantly decreased in HIV-1 HAART non-adherent compared with HIV-1 HAART adherent and healthy controls P<0.01. TNF-α and TGF-β were significantly reduced in HIV-1 HAART adherent patients at 12 months compared to those at 6 months P<0.01. IL-4 and IL-10 correlated positively with viral load. IL-4, IL-6, IL-10, TNF-α and TGF- β associated inversely with CD4 T cell counts and body mass index (BMI).

**Conclusion:**

This study established that HAART adherence is immunologically beneficial to the pro and anti-inflammatory cytokine balance milieu while non-adherence appears to cause alterations in pro and anti-inflammatory cytokines warping the balance in this dichotomy.

## Introduction

Symmetry between cytokine dichotomies is essential for regulation of the immune system^1^. Cells of the immune system secrete pro and anti-inflammatory cytokines during infection which can be assayed in serum and plasma^2^,^3^. Cytokines present in circulation are pointers to the magnitude of an immune response. CD4 T cells are the main immune cells that secrete cytokines during HIV infection^4^. These cells are usually activated and differentiate into effector subsets^5^. The classical subsets are primarily T-helper 1 (Th-1) and T-helper 2 (Th-2), but other newer subsets have been identified including follicular helper T-cell (Tfh), T-helper 17 (Th-17) and induced T-regulatory cells (iTreg) 2. Th-1 subset produces vital cytokines such as interferon- γ (IFN- γ) and interleukin 2 (IL-2) while Th-2 subset secretes IL-4, IL-5, IL-6 and IL-10 6,7. Previous studies have reported that infection with HIV down-regulates Th-1 and up-regulates Th-2 cytokines creating an imbalance and consequently impairing the immune function^8^,^9^. Therefore, in order to mount an effective cell mediated immune response, a balance between pro-inflammatory cytokines such as IL-2, IL-6, IFN- γ and tumor necrosis factor -α (TNF-α) and anti-inflammatory cytokines like IL-4, IL-5, IL-10 and tumor growth factor-β (TGF-β) is necessary.

The world health organization (WHO) recommends immediate enrollment of HIV infected patients on HAART^10^. Use of HAART has apparent benefits to HIV infected individuals like reducing viral load, opportunistic infection, preventing virologic failure and increasing life expectancy^11^–^13^. Cytokine levels in serum and plasma have been assayed in the context of HAART use. For instance, plasma IL-6 and IL-10 levels have been reported to be decreased in HAART-experienced while IL-4 remained unchanged^14^–^16^. Additionally, IFN-γ levels have been reported to be elevated in HIV patients on HAART^16^–^18^. Cellular analyses have also revealed increased IL-10 and TGF-β specific natural killer (NK) cells in patients on antiretroviral treatment^19^. Consequently, HAART restores the balance between pro- and anti-inflammatory cytokines16. Altogether, these findings imply that antiretroviral treatment modulates circulating cytokines. Nevertheless, there are very few studies that have focused on circulating levels of cytokines in non-adherent HIV patients. Therefore, it is not clear how cytokine levels are affected in HAART non-adherent HIV infected patients.

Although, there is no standard criteria for defining of non-adherence to HAART^20^,^21^. Some studies have defined HAART non-adherence as less than 95% of expected monthly drug refills for the duration of antiretroviral use^22^,^23^. Moreover, taking more than 95% of the prescribed dosage is usually considered optimal while taking less than 95% of the dosage is suboptimal^24^,^25^. The current study adopted the 95% cut off as the standard for assessing adherence. Furthermore, since cytokines act as surrogate prognostic markers in HIV patients' then quantification of circulating pro and anti-inflammatory cytokines in the context of HAART non-adherence is important for monitoring immune functions.

## Materials and methods

Study design and population. This cross-sectional study was conducted in one hundred and sixty three participants were purposively recruited at the Siaya County referral hospital HIV comprehensive care clinic in western Kenya. The study subjects consisted of 51 HIV negative control, 23 HIV-1 positive HAART -naive, 28 HIV-1 positive on first line HAART adherent for 6 months, 19 HIV-1 positive on first line HAART adherent for 12 months and 42 HIV-1 positive individuals who were non-adherent to HAART. Since the study area is a malaria and helminthes endemic site, the patients were screened for malaria and helminthes before enrolling. All the HIV-1 positive study participants were on tenofovir disoproxil fumarate (TDF) + lamivudine (3TC) + efavirenz (EFV).

### Inclusion and exclusion criteria

Study subjects were eligible if ≥18 years. Individuals with malaria, helminthes and AIDS like symptoms according to WHO criteria were excluded. In addition, those with a history of diabetes mellitus, opportunistic infections, inflammatory conditions, diarrhoea or pregnancy also were excluded. The control subjects were supposedly healthy HIV negative individuals for both determine and first response kits and were eligible if ≥18 years.

### Sample collection

3ml of whole blood were drawn by venipuncture from all participants in the study. The blood samples were aliquoted into EDTA tubes for CD4+ T cell, and viral load and serum separator tubes for cytokines. The sera were separated and stored at ≤ -20 °C until analyses.

### CD4 T cell enumeration

CD4+ T cell counts were determined in an automated fashion using the BD FACSCaliburTM flow cytometer (Becton-Dickinson™, Franklin Lakes, USA). Briefly, 5.0 µl of EDTA blood samples were placed in a tube and RBC lysis buffer added. After 5 minute incubation, the cells were washed and fluorescent-tagged antibodies (anti-CD3, anti-CD4, and anti-CD45) were added. The cells were incubated for 30 minutes after which the samples were washed and the CD4+ T cells enumerated on the flow cytometer.

### HIV-1 viral load

HIV-1 viral loads were determined using the automated Abbott m2000 System according to the manufacturer's instructions (Abbott Molecular Inc., Illinois, USA). Briefly, RNA was extracted from 0.2 ml serum samples and reverse-transcribed into cDNA. The cDNA was amplified using HIV-1-specific and internal control primers. Fluorescence intensity of the HIV-1 probe was converted into viral loads by the analyzer.

### Cytokine measurements

Circulating levels of IL-4, IL-6, IL-10, IFN-γ, TNF-α and TGF-β were determined in serum samples using a sandwich enzyme linked immunosorbent assay (ELISA) according to the manufacturer's protocols (R&D Systems, Inc., Minneapolis, USA).

### Data analysis

Statistical analysis was conducted using IBM® SPSS Statistics 23.0 (SPSS Inc. Chicago, USA). Continuous data (age, and laboratory measures) summarized as medians (IQR) and categorical data presented as proportions were tabulated. Differences in the proportions were determined using the chi-square tests. Statistical comparisons of the continuous data across the study groups were performed using non-parametric ANOVA (Kruskal Wallis) tests followed by Bonferroni's posthoc for multiple comparisons test with a cut off set at P<0.01. Spearman's Rank correlation analyses were used to determine the associations of cytokine levels with viral load, CD4 T cell count and BMI. All tests were two-tailed and P<0.05 was used for statistical inferences.

### Ethical consideration

Ethical approval was obtained from the Masinde Muliro University of Science and Technology Institutional Ethics Review Committee (Protocol: MMU/COR-403012-V39) and permission conduct the study at the comprehensive care clinic from the Siaya County referral hospital management. Written informed consent was obtained from the study participants prior to enrolment into the study. All HIV-1 infected HAART -naive patients were immediately enrolled on first line treatment of TDF + 3TC + EFV. Information collected as part of this study was kept confidential unless required for patient care, with only access limited to the investigators.

## Results

Demographic and clinical profiles of the study participants. The demographic and clinical characteristics of the study participants are summarized in Table 1. A total 163 adults (males, n=73 and females, n=90) were recruited to the study. Age (P=0.875), gender (P=0.109) and height (P=0.634) were similar across the five clinical arms. Body weight (P<0.0001), BMI (P<0.0001) and CD4 T cell counts (P<0.0001) varied across all the study groups. Similarly, viral load (P<0.0001) was significantly different across four clinical groups since the control arm were HIV negative. Post-hoc analyses using Bonferroni correction were done to reveal between group differences and a cut-off of P<0.01 set. The main outcome group HIV-1 + HAART+ non-adherent (median, median, 59.1; IQR, 11.8 kg) had significantly lower median body weight than controls (median, 67.8; IQR, 18.0 kg) and HIV-1 + HAART + with 12 months use (median, 73.4; IQR, 18.2 kg) P<0.01 participants. These analyses revealed no difference in median body weight between HIV-1 + HAART naive and HIV-1 + HAART non-adherent, P>0.01. Additionally, HIV-1 + HAART + non-adherent had lower BMI compared with HIV-1 - HAART- (median, 21.1; IQR 3.7 kg/m^2^ vs median, 24.5; IQR 5.3 kg/m2; P<0.01) and HIV-1 + HAART + with 12 months use (median, 21.1; IQR 3.7 kg/m^2^ vs median, 25.8; IQR 7.2 kg/m^2^; P<0.01). HIV-1 + HAART+ non-adherent (median, 423; IQR 252 cells/µl) had significantly lower CD4 T cell counts compared with healthy controls (median, 1407; IQR 1303 cells/µl), P>0.01.

**Table 1:** Socio-demographic and clinical characteristics of the study participants

Characteristics	HIV-HAART -, n=51	HIV-1+ HAART-, n=23	HIV-1+ HAART+, 6 months use, n=28	HIV-1+ HAART+, 12 months use, n=19	HIV-1+ HAART+, non-adherent, n=42	*P*
Age, yrs.	37.0 (6.0)	38.0 (7.0)	37.0 (6.8)	37.0 (8.0)	36.5 (9.3)	0.875
Female/Male, n (%)	27/24 (52.9/47.1)	14/9 (60.9/39.1)	20/8 (71.4/28.6)	12/7 (63.2/36.8)	17/25 (40.5/59.5)	0.109
Weight, kg	67.8 (18.0)^a^	59.9 (11.0)	63.7 (12.7)	73.4 (18.2)^a^	59.1 (11.8)	**<0.0001**
Height, m	1.7 (0.1)	1.7 (0.1)	1.7 (0.1)	1.7 (0.1)	1.7 (0.1)	0.634
BMI, kg/m^2^	24.5 (5.3)^a^	20.4 (5.1)	23.4 (4.1)	25.8 (7.2)^a^	21.1 (3.7)	**<0.0001**
<18.5 kg/m^2^ n (%)	4 (21.1)	3 (15.8)	3 (15.8)	0 (0.0)	9 (47.4)	-
≥18.5 kg/m^2^ n (%)	47 (32.6)	20 (13.9)	25 (17.4)	19 (13.2)	33 (22.9)	-
Log_10_HIV-1 RNA copies/ml	-	4.5 (1.6)	3.1 (1.7)	3.6 (1.9)	4.6 (1.1)	**<0.0001**
<1000 copies/ml n (%)	0 (0.0)	4 (5.1)	13 (16.7)	7 (9.0)	3 (3.8)	-
≥ 1000 copies/ml n (%)	0 (0.0)	19 (22.4)	15 (17.6)	12 (14.1)	39 (45.9)	-
CD4+ T cells/µl	1407 (1303)^a^	396 (349)	577 (357)	527 (508)	423 (252)	**<0.0001**
<500 cells/µl n (%)	3 (4.5)	16 (23.9)	9 (13.4)	9 (13.4)	30 (44.8)	-
≥500 cells/µl n (%)	48 (50.0)	7 (7.3)	19 (19.8)	10 (10.4)	12 (12.5)	-

Data are presented as medians (IQR, interquartile range) or as indicated. Data analysis was conducted using Kruskal-Wallis H test across groups with *post-hoc* Bonferroni correction between group comparison set at *P*<0.01 for continuous measures and Chi-Square test for gender distribution. BMI, body mass index.

a *P*<0.01 *vs* HIV+ HAART+ non-adherent. Values in bold are significnt values.

Serum cytokine profiles of study participants. The serum cytokine levels of the study subjects are shown in Table 2. All the six cytokines assayed in the current study showed significant differences in serum levels across the study groups. Similarly, post-hoc analyses using Bonferroni correction were done to establish between group differences and a cut-off of P<0.01 set. IL-4 levels were higher in HIV-1 + HAART + non-adherent (median, 22.1; IQR 8.6 pg/ml) compared with HIV-1 - HAART- (median, 3.7; IQR 3.8 pg/ml), HIV-1 + HAART + with 6 months use (median, 9.5; IQR 4.4 pg/ml) and HIV-1 + HAART + with 12 months use (median, 3.2; IQR 2.1 pg/ml). Conversely, HIV-1 + HAART + non-adherent had lower IL-4 levels compared with HIV-1+ HAART - naive (median, 29.9; IQR 8.6 pg/ml). Similarly, HIV-1 + HAART + non-adherent participants presented with significantly elevated serum IL-6, IL-10 and TNF-α when compared with HIV-1 - HAART-, HIV-1 + HAART + with 6 months use and HIV-1 + HAART + with 12 months use, P<0.01. However, these cytokines were significantly higher in HIV-1 + HAART – naive when compared with HIV-1 + HAART + non-adherent. IFN- γ levels were significantly decreased among HIV-1 + HAART + non-adherent compared with HIV-1 - HAART-, HIV-1 + HAART + with 6 months use and HIV-1 + HAART + with 12 months use, P<0.01. IFN- γ levels in HIV-1 + HAART- naïve were comparable with HIV-1 + HAART + non-adherent. <=>TGF-β were significantly increased in HIV-1 + HAART + non-adherent subjects likened to HIV-1 - HAART- and HIV-1 + HAART + with 12 months use, P<0.01.

**Table 2:** Serum cytokine levels of the study participants

Cytokine, pg/ml	HIV-HAART -, n=51	HIV+ HAART-, n=23	HIV+ HAART+, 6 months use, n=28	HIV+ HAART+, 12 months use, n=19	HIV+ HAART+, non- adherent, n=42	*P*
**IL-4**	3.7 (3.8)^a^	29.9 (8.6)^a^	9.5 (4.4)^a^	3.2 (2.1)^a^	22.1 (8.6)	**<0.0001**
**IL-6**	4.7 (1.1)^a^	16.9 (5.7)^a^	9.8 (5.9)^a^	8.2 (3.5)^a^	13.4 (7.6)	**<0.0001**
**IL-10**	5.4 (1.9)^a^	32.2 (7.2)^a^	19.5 (7.0)^a^	7.2 (2.2)^a^	27.2 (9.7)	**<0.0001**
**IFN-γ**	4.9 (1.8)^a^	0.7 (0.1)	2.3 (1.0)^a^	3.1 (0.6)^a^	0.9 (0.7)	**<0.0001**
**TNF-α**	9.4 (0.8)^a^	38.7 (17.1)^a^	29.9 (12.0)	18.6 (5.9)^a^	29.8 (17.7)	**<0.0001**
**TGF-β**	34.4 (10.0)^a^	89.3 (29.1)	77.7 (41.4)	64.3 (26.3)^a^	81.1 (35.6)	**<0.0001**

Data are presented as medians (IQR, interquartile range). Data analysis was conducted using Kruskal-Wallis H test across groups with *post-hoc* Bonferroni correction between group comparison set at *P*<0.01 for continuous measures.

a *P*<0.01 vs. HIV+ HAART+ non-adherent. Values in bold are significantalues.

Association of circulating cytokine levels with HIV clinical markers. Association of cytokine levels with clinical markers is shown in Table 3. IL-4 correlated positively with viral load (ρ= 0.409; P<0.0001) while it correlated inversely with CD4 T cell count (ρ= -0.521; P<0.0001) and BMI (ρ= -0.502; P<0.0001). Similarly, IL-10 correlated positively with viral load (ρ= 0.272; P=0.004) and inversely with CD4 T cell count (ρ= -0.627; P<0.0001) as well as BMI (ρ= -0.376; P<0.0001). IL-6 and TNF-α correlated inversely with CD4 T cell count (ρ= -0.492; P<0.0001 and ρ= -0.595; P<0.0001) and BMI (ρ= -0.308; P<0.0001) and (ρ= -0.292; P<0.0001) respectively. Conversely, IFN-γ correlated inversely with viral load (ρ= -0.326; P<0.0001) and positively with CD4 T cell count (ρ= 0.619; P<0.0001) in addition to BMI (ρ= -0.342; P<0.0001). TGF-β correlated negatively with CD4 T cell counts (ρ= -0.526; P<0.0001) and BMI (ρ= -0.216; P=0.006).

**Table 3:** Association of serum cytokine levels with HIV prognostic markers

*Cytokine*	Log_10_ HIV-1 RNA copies		CD4+ T cells		BMI	
	ρ	*P*	ρ	*P*	ρ	*P*
***IL-4***	0.409	**<0.0001**	-0.521	**<0.0001**	-0.502	**<0.0001**
***IL-6***	0.129	0.175	-0.492	**<0.0001**	-0.308	**<0.0001**
***IL-10***	0.272	**0.004**	-0.627	**<0.0001**	-0.376	**<0.0001**
***IFN-γ***	-0.326	**<0.0001**	0.619	**<0.0001**	0.342	**<0.0001**
***TNF-γ***	0.139	0.145	-0.595	**<0.0001**	-0.292	**<0.0001**
***TGF-β***	-0.081	0.395	-0.526	**<0.0001**	-0.216	**0.006**

Data presented are correlation coefficient (rho, ρ) with associated values. Statistical analysis was performed using Spearman's rank correlation test. IL-4, interleukin-4. IL-6, interleukin-6. IL-10, interleukin-10. IFN-γ, interferon-gamma. TNF-α, tumor necrosis factor-γ. TGF-β, tumor growth factor-β. Values in bold indicate significant *P* values.

Predictors of HIV disease markers. Regression modelling for cytokines predicting viral load in HIV infected individuals was significant (F (6, 162) =21.216, P<0.0001) with the entire set of cytokines (IL-4, IL-6, IL-10, IFN-γ, TNF-α and TGF-β) accounting for 42.9% of the variance in the viral load (R=0.671, R2=0.429) while IFN-γ individually influenced viral load by 13.5%, P<0.0001. The other cytokines did not have significant influence on the viral load. Prediction for CD4 T cell counts in HIV infected participants was also significant (F (6, 162) =21.504, P<0.0001) with all the six cytokine accounting for 41.5% variation in CD4 T cell counts (R=0.661, R2=0.415). IFN-γ affected CD4 T cell counts significantly by 2.7%, P=0.004. Overall variance in BMI was significant (F (6, 162) =8.497, P<0.0001) with the cytokines assayed accounting for 19.3% of the variance. IL-4 is the only cytokine that had individual significant influence on BMI by 4.8%, P=0.002.

## Discussion

The current study assayed pro and anti-inflammatory cytokines in HIV-1 infected patients who were non-adherent to HAART and compared the serum levels with HAART adherent, naive as well as healthy controls. The pro-inflammatory cytokines analyzed included IL-6, IFN-γ and TNF-α. IL-6 and TNF-α were elevated in HIV-1 infected individuals and decreased with consistent HAART use. IL-6 and TNF-α appeared to decrease steadily in patients who were on HAART regularly. Nevertheless, IL-6 and TNF-α were found to be elevated in non-adherent patients. On the other hand serum IFN-γ levels were decreased in HIV-1 infected patients. Consistent use of HAART seemed to lead to an elevation of serum IFN-γ. Non-adherence seemed to cause a decrease in serum IFN-γ levels. Contrary to these findings, some studies have reported no changes in serum IL-6 levels among HIV patients on HAART^26^,^27^. While a recent finding showed that IL-6 in HIV patients on HAART were likely to increase when CD4 T cells increased14. Though, several longitudinal reports have revealed concordant findings with the current study of a decrease in IL-6 with adherence to HAART^16^,^28^. TNF-α has also been found to be elevated in HIV infected HAART naive individuals but serum levels decrease with HAART use^29^,^30^. Although, it is important to note that different HAART regimens affect TNF-α levels differently^31^. Also, there seems to be no clear pattern on how HAART influences serum IFN-γ levels in HIV. Some studies have reported constantly high circulating IFN-γ levels even with consistent HAART use^18^,^32^. Conversely, some studies find IFN-γ levels to be decreased in HIV infected individuals on HAART^16^,^33^. This study reports an elevation in serum IFN-γ levels with adherence to HAART. IFN-γ is a Th1 cytokine, usually infection with HIV causes a shift from Th1 to Th2^34^,^35^. Accordingly, serum IFN-γ are expected to be low during infection while HAART use should boost the secretion of IFN-γ by immune cells. Overall, these pro-inflammatory cytokines play important immune roles during infection with HIV-1. Our findings appear to suggest a reversal on serum cytokine levels following non-adherence. IL-6 and TNF-α were elevated while IFN-γ decreased in individuals who were non-adherent HIV-1 interfering with the effect of HAART on circulating pro-inflammatory cytokines. Furthermore, this study established a positive correlation between IL-4 and viral load. Likewise, IFN-γ correlated positively with CD4 T cell counts and BMI. Contrariwise, IL-4, TNF-α and IFN-γ associated inversely with CD4 T cell counts and BMI. Similarly other studies have reported elevated IL-4 levels in HIV patients with a high viral load^36^. IFN-γ being a Th1 cytokine is definitely down-regulated during HIV-1 infection promoting an environment suitable for viral replication and CD4 T cell loss^37^. This isconsistent with hierarchical analysis in this study that revealed IFN-γ to have an overall influence on viral load and CD4 T cell counts.

IL-4, IL-10 and TGF-β were the anti-inflammatory cytokines examined in this study. Infection with HIV seemed to cause an elevation of serum levels of the three cytokines. Adherence to HAART over a long duration appeared to lower anti-inflammatory cytokines in circulation. However, circulating levels of these cytokines in non-adherent HIV patients were found to be raised. Infection with HIV has been shown to lead to a switch from Th1 to Th2 cytokines where IL-4 and IL-10 belong^38^,^39^. Consequently, these cytokines are expected to be elevated in serum after infection. Since HAART acts to restore cytokine balance, it follows that consistent use should lower serum IL-4 and IL-10 significantly8. However, a recent study comparing serum IL-4 and IL-10 levels in HAART naive and experienced HIV patients found similar IL-4 levels while IL-10 was decreased significantly in HAART experienced individuals^40^. TGF-β in cellular immunity has been shown to block Th1/Th2 differentiation and suppresses IL-2, IL-4 and IFN-γ secretion^41^,^42^. Elevated serum levels have been consistently seen after infection with HIV indicative of disease progression^43^. Appropriate use of HAART lowers serum TGF-β levels significantly improving disease prognosis^44^. In the current study, total adherence to HAART seemed to lower serum levels of the anti-inflammatory cytokines in effect improving disease outcomes in HIV-1 patients. Though, non-adherence appeared to cause an elevation in anti-inflammatory cytokines thus reversing the effects of HAART. Additionally, IL-4 and IL-10 were positively correlated with viral load while IL-4, IL-10 and TGF-β were inversely associated with CD4 T cell counts and BMI. These findings imply adverse effects off non-adherence to HAART since non-adherence caused elevation of anti-inflammatory cytokines. Elevated IL-4 and IL-10 for instance is correlated with high viral load, low CD4 T cells and BMI. In addition, hierarchical prediction showed IL-4 to have an influence on BMI in HIV-1 infected patients in addition to all the cytokines combined. Raised TGF-β would result in low CD4 T cells and BMI hence poor prognosis. Studies have shown that HAART causes secretion of IL-2 which restores Th1 cytokine profiles in HIV individuals^45^. Therefore, non-adherence may distort Th1 cytokine restoration causing an imbalance in the pro/anti-inflammatory cytokine milieu.

Overall, this study revealed that non-adherence to HAART results in the elevation of serum IL-4, IL-6, IL-10, TNF-α and TGF-β while it decreases IFN-γ. These findings suggest a reversal of the effects of adherence to HAART and modulation of the Th1/ Th2 cytokine switch. This ultimately leads to adverse effects and poor HIV-1 prognosis. Also, IFN-γ I and IL-4 are important predictors of markers ofdisease progression in HIV-1 infected non-adherent individuals.

## Conclusion

This study found out that non-adherence to HAART in HIV-1 infected individuals alters serum levels of pro (TNF-α, IL-6 and IFN-γ) and anti-inflammatory (IL-4, IL-10 and TGF-β) cytokines, distorting the balance in this dichotomy. Additionally, circulating IL-6, TNF-α, IFN-γ, IL-4, IL-10 and TGF-β levels can be used as potential surrogate markers of HIV-1 disease progression and antiretroviral use. Likewise, to monitor disease prognosis in HIV-1 infected HAART non-adherent individuals.

## Recommendation

In our view, additional studies with greater number of recruited participants is required to validate the current findings over time.

## Data Availability

The data used to support the findings of this study are included within the article.

## Notes

**Declarations**
